# Maternal age and blastocyst morphology as independent predictors of embryonic euploidy in preimplantation genetic testing cycles: A retrospective cohort study

**DOI:** 10.1097/MD.0000000000049126

**Published:** 2026-06-05

**Authors:** Hui Huang, Fengxia Liu, Kali Huang, Li Deng, Xuefei Liang, Xueyuan Liu

**Affiliations:** a Department of Reproductive Medicine, The Reproductive Hospital of Guangxi Zhuang Autonomous Region, Nanning, Guangxi, China.

**Keywords:** blastocyst, euploidy, maternal age, morphology, preimplantation genetic testing, trophectoderm

## Abstract

In this study, we aimed to investigate which clinical/embryological factors function as independent predictors of embryonic euploidy in preimplantation genetic testing cycles. This retrospective cohort study included 377 couples with normal parental karyotypes undergoing their first preimplantation genetic testing cycle, yielding 1105 embryos for analysis. The primary outcome was embryonic euploidy. Associations were evaluated using multivariable generalized linear mixed-effects models with patient-level random intercepts. Maternal age and blastocyst morphology emerged as independent predictors of euploidy. Each additional year of maternal age reduced the odds of euploidy (adjusted odds ratio [aOR]: 0.88, 95% confidence interval [CI]: 0.84–0.93; *P* < .001). Better trophectoderm morphology was strongly associated with euploidy (Grade B: aOR 2.43, 95% CI: 1.71–3.46; Grade A: aOR 4.42, 95% CI: 2.78–7.04; both *P* < .001). Similarly, Grade A inner cell mass showed a significant independent association (aOR: 1.69, 95% CI: 1.01–2.82; *P* = .045), while Grade B was borderline (*P* = .065). Anti-Müllerian hormone showed a modest independent association (aOR: 1.10 per ng/mL, 95% CI: 1.01–1.20; *P* = .033), while estradiol on trigger day was statistically significant but with negligible effect size (aOR: 0.99; *P* = .025); therefore, these parameters were considered of limited clinical relevance. Maternal age and blastocyst morphology, specifically trophectoderm and inner cell mass grades, were robust independent predictors of euploidy, with higher morphological grades associated with higher euploidy rates. These findings support morphology- and age-based embryo selection strategies.

## 1. Introduction

Assisted reproductive technology (ART) has become a cornerstone of infertility treatment worldwide; however, its success fundamentally depends on the transfer of chromosomally normal (euploid) embryos. Aneuploidy is highly prevalent in preimplantation human embryos, affecting more than half of all cases. It represents a major cause of implantation failure, biochemical pregnancy loss, and miscarriage, and its incidence rises markedly with advancing maternal age.^[1]^ These adverse outcomes impose substantial clinical and personal burdens, including repeated procedures, treatment delays, and financial costs. Preimplantation genetic testing (PGT) is increasingly routine, enhancing embryo selection by directly assessing embryonic chromosomal status prior to transfer. Over the past decade, PGT use has expanded steadily, and multiple clinical series have reported improved reproductive outcomes in selected populations.^[2]^ By identifying euploid embryos, PGT for aneuploidy (PGT-A) facilitates the investigation of determinants of euploidy and the steps within the ART pathway that may influence euploidy rates. Such insights are clinically meaningful for optimizing ART protocols, improving pregnancy outcomes, guiding clinician–patient counseling, and informing embryo transfer strategies.

A broad range of factors has been examined, including clinical characteristics, controlled ovarian hyperstimulation (COH) protocols and responses, and embryological laboratory metrics. Although several of these factors have been reported as independent determinants of euploidy,^[3–5]^ conclusions across studies remain inconsistent. Among maternal factors, increasing age and body mass index are associated with an increased risk of aneuploidy, whereas evidence regarding ovarian reserve markers and stimulation procedures remains mixed.^[6–11]^ Paternal factors associated with euploidy include age, sperm concentration, and motility.^[12,13]^ Aneuploidy rates vary widely across centers (39.5%–82.5%), suggesting substantial center-level variability that may reflect differences in stimulation strategies, laboratory processes, or testing platforms.^[14]^ However, causal inference is limited by confounding factors.

Embryonic morphology and developmental kinetics are classic indicators of embryo quality, but their ability to predict euploidy remains controversial. The Gardner grading system, which assesses blastocyst expansion, inner cell mass (ICM) quality, and trophectoderm (TE) quality, is the most widely used standardized framework for blastocyst evaluation.^[15]^ Both the ICM and TE are critical for postimplantation development, with the ICM forming the fetus and the TE forming the placenta and extraembryonic tissues. Higher ICM and TE grades have been associated with increased euploidy rates.^[16]^ Conversely, slower-developing blastocysts exhibit higher aneuploidy rates, potentially reflecting meiotic spindle abnormalities, mitochondrial dysfunction, or aberrant gene expression.^[17]^ Nonetheless, morphology and kinetics are only partially correlated with euploidy, and implantation failure can occur even in high-grade, rapidly developing blastocysts.^[18]^

Despite these insights, significant limitations persist in the existing evidence base. Many studies are single-center, cross-sectional, or embryo-level analyses that do not account for the hierarchical structure of ART data, in which observations from the same patient or cycle are correlated. Without accounting for within-patient and within-cycle clustering, effect estimates may be biased or overly precise. Additionally, the near-universal restriction to PGT cycles introduces selection and indication biases and may amplify between-center heterogeneity resulting from variations in testing platforms and laboratory workflows. These issues underscore the need for robust statistical methods and sufficiently large, well-characterized cohorts.

In this retrospective cohort study, we leveraged clinical and embryological data from our center to identify independent predictors of embryonic euploidy in PGT cycles. By applying generalized linear mixed-effects models (GLMMs) to account for patient-level clustering in a well-powered cohort, we aimed to provide robust evidence to enhance clinician–patient counseling, optimize ART workflows, and ultimately improve the likelihood of healthy live births. While aneuploidy reflects biological errors in gametogenesis or embryogenesis, we selected euploidy as the primary outcome because it represents the direct, clinically actionable endpoint required for embryo transfer and subsequent healthy live birth.

## 2. Methods

**Figure 1.**
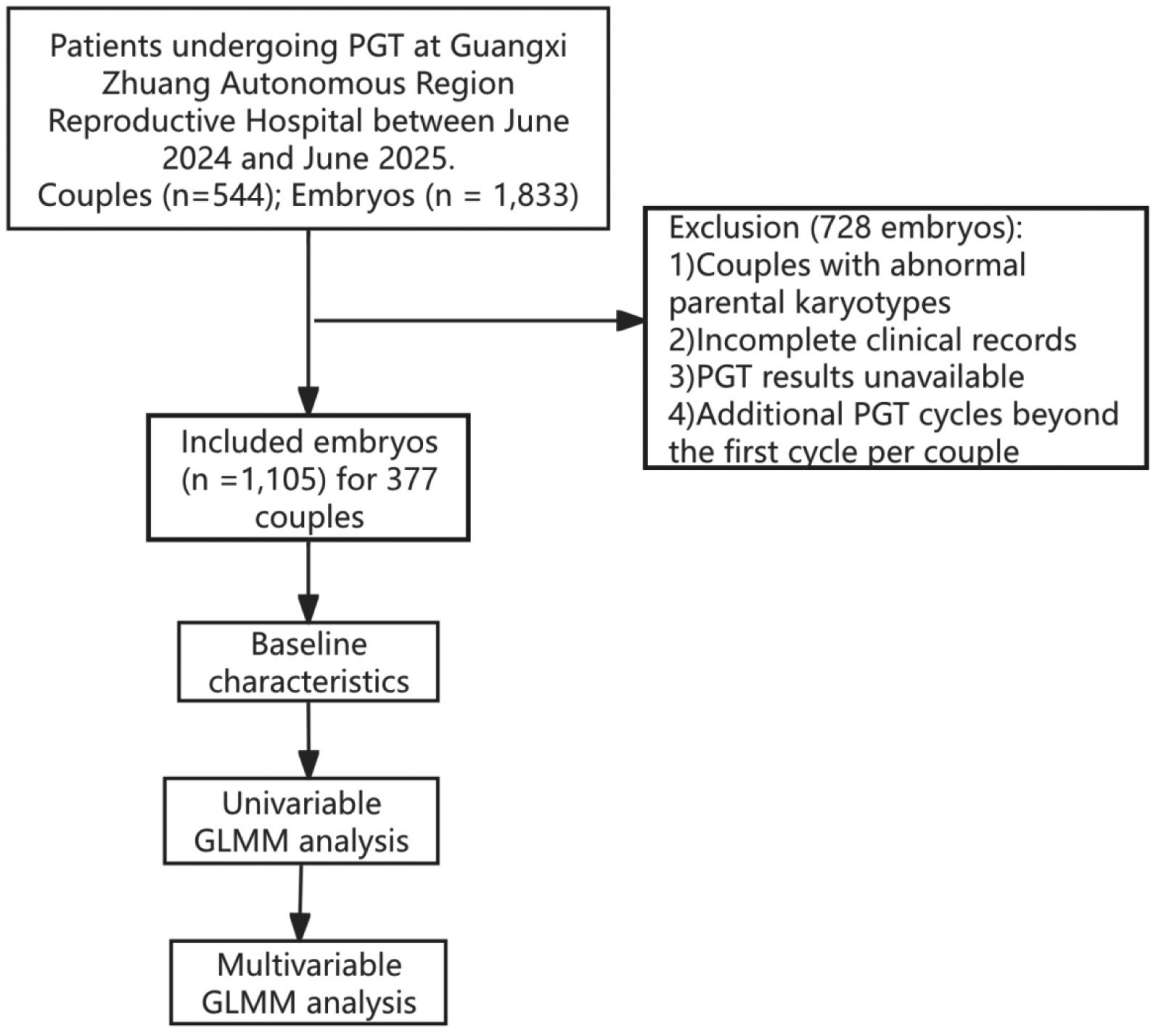
The flow diagram of the study. GLMM = generalized linear mixed-effects model, PGT = preimplantation genetic testing.

We retrospectively analyzed the clinical data of patients who underwent PGT at the Guangxi Zhuang Autonomous Region Reproductive Hospital between June 2024 and June 2025. Only the first PGT cycle per couple was included, and both partners were required to have normal karyotypes. Cases were excluded if either partner had a chromosomal abnormality, if patient records were incomplete, or if PGT results were unavailable. Initially, a total of 1833 embryos from 544 couples undergoing PGT within the study period were identified. After applying the inclusion and exclusion criteria, a total of 1105 embryos from 377 couples were included in the final cohort. The flow diagram is presented in Figure 1. A formal sample size calculation was not conducted before study initiation because the cohort comprised all eligible cycles within the predefined study period. Instead, a post hoc power assessment was carried out to confirm that the study had sufficient statistical power.

This study was approved by the Ethics Committee of the Reproductive Hospital of Guangxi Zhuang Autonomous Region (No. KY-LW-2025-18), and the requirement for informed consent was waived due to the retrospective nature of the study. The study design and reporting adhered to the Strengthening the Reporting of Observational Studies in Epidemiology (STROBE) guidelines.

### 2.1. Data collection

Baseline characteristics, types of clinical protocol, and ART laboratory parameters were collected. The variables were classified as numerical or categorical (see Table S1, Supplemental Digital Content). The primary outcome was embryonic euploidy.

### 2.2. Clinical protocol

Fresh stimulation protocols were personalized according to patient characteristics and implemented in accordance with the 2019 ESHRE ovarian stimulation guidelines.^[19]^ Gonadotropin initiation was individualized, and the dose was adjusted based on the ovarian response of each patient. The trigger day was determined based on the follicular size, together with serum estradiol and progesterone levels. Final oocyte maturation was induced using either human chorionic gonadotropin (hCG) combined with recombinant hCG or a gonadotropin-releasing hormone agonist in conjunction with hCG, according to the clinician’s protocol and patient response. Oocyte retrieval was performed 34 to 38 hours after triggering with transvaginal ultrasound guidance. Mature oocytes at metaphase II (MII) were fertilized by intracytoplasmic sperm injection and cultured in vitro until the blastocyst stage.

### 2.3. Embryo assessment

Day 3 embryos were graded using a 4-grade morphological system that evaluated the blastomere number, homogeneity, and percentage of fragmentation (Alpha Scientists in Reproductive Medicine and ESHRE Special Interest Group of Embryology, 2011). Grades I and II represented high quality (uniform blastomeres, <20% fragmentation), whereas Grades III and IV captured progressively poorer morphology, characterized by increased fragmentation, irregularity, or degeneration. Embryos with at least 7 cells and Grades I and II morphology were classified as high quality.

Day 5 or 6 blastocysts were evaluated using the Gardner scoring system to evaluate the developmental stage (1–6), ICM quality (A–C), and TE quality (A–C).^[15]^ Representative images of blastocysts with different morphological grades are shown in Supplemental Digital Content 2. ICM and TE grades were assigned only to blastocysts at stages 3 to 6 with clearly distinguishable components. High-quality blastocysts were defined as those graded 3BB or higher.

### 2.4. PGT analysis

A blastocyst biopsy was performed on day 5, 6, or 7 for blastocysts graded AA, AB, BA, BB, BC, CB, AC, or CA. The chromosomal status of each sample was determined using next-generation sequencing. Euploidy was defined as the presence of 46 normal chromosomes.

### 2.5. Statistical analysis

Analyses were conducted using complete-case data; all variables included in the analyses were complete, and no imputation was required in the final study cohort.

Statistical analyses were performed using R software (version 4.3.1; The R Foundation for Statistical Computing, Vienna, Austria). All statistical tests were two-sided, and *P* < .05 was considered statistically significant. Baseline characteristics of the euploid and aneuploid groups were compared using the gtsummary package (version 2.3.0; CRAN [The Comprehensive R Archive Network]) in R. For numerical variables, distributional assumptions were assessed in each group using the Shapiro–Wilk test. Variables that approximated normality in both groups are expressed as means ± standard deviations, and group differences were evaluated using the Welch *t* test. When normality was not supported, values are summarized as medians (Q1; Q3) and compared using the Wilcoxon rank-sum (Mann–Whitney *U*) test. Categorical variables are presented as counts and percentages, with between-group differences assessed using the chi-square test or Fisher exact test when the expected cell counts were small (<5).

To account for the clustering of multiple embryos within patients, embryo-level GLMMs with a logit link were fitted using the lme4 package (version 1.1.37; CRAN [The Comprehensive R Archive Network]) in R, incorporating a random intercept for each patient.

For the univariable analysis, each candidate predictor was selected based on a priori evidence, clinical interpretability, and statistical feasibility; these were subsequently evaluated in separate GLMMs to identify the factors associated with embryonic euploidy. Predictors that satisfied the screening threshold (*P* < .1 in univariable analysis), along with prespecified clinically important covariates, were included in a multivariable GLMM. Backward elimination was applied to obtain a parsimonious model, retaining variables with *P* < .05. Results are reported as adjusted odds ratios (aORs) with corresponding 95% confidence intervals (CIs). From the multivariable model, the adjusted effects were presented using a forest plot. Additionally, plots were generated to illustrate the association between female age and euploidy rate, as well as the associations between various predictors and euploidy rate across predefined age strata.

## 3. Results

The overall euploidy rate was 48.1% (531/1105). Several significant differences in baseline characteristics were observed between the euploid (n = 531) and aneuploid (n = 574) embryo groups (Table 1). The euploid group was characterized by significantly younger female patients (median age: 38 vs 40 years; *P* < .001) and younger male partners (median age: 38 vs 41 years; *P* < .001). Additionally, the euploid group demonstrated better ovarian reserve parameters, including higher anti-Müllerian hormone (AMH) levels (2.94 vs 2.04 ng/mL, *P* < .001), and superior cycle outcomes, reflected by a greater number of retrieved oocytes (14 vs 12, *P* < .001), MII oocytes (11 vs 9, *P* < .001), and embryos available for biopsy (4 vs 3, *P* < .001). Although most sperm parameters showed minimal differences, the euploid group had a slightly lower sperm concentration (*P* = .003) but better sperm quality, as evidenced by higher rates of morphologically normal sperm (*P* = .006) and lower DNA fragmentation index (DFI; *P* = .002). Notably, the embryo quality parameters strongly favored the euploid group, which showed significantly higher proportions of high-quality blastocysts (60% vs 36%, *P* < .001), better TE grades (Grade A: 22% vs 10%, *P* < .001), and more favorable ICM grades (Grade A: 19% vs 11%, *P* < .001). The distribution of COH protocols was comparable between the groups; however, the euploid group had a higher proportion of cycles of PGT for monogenic disorders (PGT-M) (33% vs 14%, *P* < .001).

**Table 1:** Baseline characteristics by ploidy status of the embryos.

Characteristic	Euploid group (n = 531)	Aneuploid group (n = 574)	*P*-value
Female age (yr)	38.00 (32.00–40.00)	40.00 (38.00–42.00)	<.001*
Female BMI (kg/m^2^)	22.27 (20.63–24.84)	22.70 (20.89–24.97)	.13
Duration of infertility (yr)	2.00 (0.90–5.00)	2.50 (1.00–6.00)	.004*
AMH (ng/mL)	2.94 (1.67–4.43)	2.04 (1.13–3.51)	<.001*
Basal FSH (IU/L)	6.99 (5.89–8.24)	6.86 (5.92–8.11)	.9
Male age (yr)	38.00 (34.00–41.00)	41.00 (37.00–44.00)	<.001*
Male BMI (kg/m^2^)	24.22 (22.39–26.81)	24.51 (22.59–26.67)	.2
Sperm concentration (×10^6^/mL)	40.00 (19.90–67.30)	43.75 (25.50–76.70)	.003*
Sperm viability (%)	49.40 (34.20–61.60)	49.00 (34.70–63.30)	.4
Progressive motility	39.20 (25.70–50.30)	39.80 (25.70–51.80)	.4
Nonprogressive motility	8.50 (6.00–11.90)	9.00 (6.50–12.50)	.066
Morphologically normal sperm (%)	4.00 (3.00–6.00)	4.00 (3.00–6.00)	.006*
Sperm DNA fragmentation index (%)	12.00 (5.70–18.10)	13.70 (7.60–20.30)	.002*
E_2_ on hCG trigger day (pg/mL)	3197.00 (2182.00–4434.00)	2896.00 (1823.00–4279.00)	.002*
Number of oocytes	14.00 (10.00–22.00)	12.00 (7.00–18.00)	<.001*
Number of MII oocytes	11.00 (8.00–16.00)	9.00 (6.00–14.00)	<.001*
Number of biopsied embryos	4.00 (3.00–7.00)	3.00 (2.00–5.00)	<.001*
COH protocol			.8
Long protocol	278 (52%)	297 (52%)	
Antagonist protocol	253 (48%)	277 (48%)	
PGT type			<.001*
PGT-A	358 (67%)	492 (86%)	
PGT-M	173 (33%)	82 (14%)	
Cleavage-stage embryo quality			.5
Low quality	138 (26%)	159 (28%)	
High quality	393 (74%)	415 (72%)	
Biopsied blastocyst quality			<.001*
Low quality	212 (40%)	365 (64%)	
High quality	319 (60%)	209 (36%)	
Inner cell mass grade			<.001*
C	84 (16%)	113 (20%)	
B	347 (65%)	400 (70%)	
A	100 (19%)	61 (11%)	
Trophectoderm grade			<.001*
C	129 (24%)	251 (44%)	
B	287 (54%)	268 (47%)	
A	115 (22%)	55 (10%)	
Biopsied blastocyst stage			.033*
D5	180 (34%)	156 (27%)	
D6	334 (63%)	391 (68%)	
D7	17 (3%)	27 (5%)	

AMH = Anti-Müllerian hormone, BMI = body mass index, COH = controlled ovarian hyperstimulation, E_2_ = estradiol, FSH = follicle-stimulating hormone, hCG = human chorionic gonadotropin, MII = metaphase II, PGT = preimplantation genetic testing, PGT-A = preimplantation genetic testing for aneuploidy, PGT-M = preimplantation genetic testing for monogenic disorders.

* *P* < 0.05.

Univariable GLMM analysis identified multiple factors that were significantly associated with embryonic euploidy (Table 2). Among female-related factors, advanced maternal age substantially reduced the likelihood of euploidy (odds ratio [OR]: 0.85, 95% CI: 0.82–0.87; *P* < .001), whereas better ovarian reserve markers, such as higher AMH levels, were associated with increased odds (OR: 1.19, 95% CI: 1.10–1.28; *P* < .001). Among male-related factors, each additional year of paternal age was associated with lower odds of euploidy (OR: 0.90, 95% CI: 0.88–0.93; *P* < .001); furthermore, increasing DFI (OR: 0.98 per 1% increase, 95% CI: 0.97–0.99; *P* = .008) and lower proportions of morphologically normal sperm (OR: 0.92 per 1% decrease, 95% CI: 0.86–0.99; *P* = .018) were associated with a decreased likelihood of euploidy. Cycle performance indicators consistently predicted better outcomes, including estradiol concentrations on the hCG trigger day (OR: 1.01, *P* < .1) as well as greater numbers of oocytes retrieved (OR: 1.05, *P* < .001), MII oocytes (OR: 1.07, *P* < .001), and biopsied embryos (OR: 1.23, *P* < .001). Notably, embryological parameters showed particularly strong associations: superior TE grades (*P* < .001) and ICM grades (*P* < .001) were associated with significantly increased odds of euploidy. Additionally, PGT-M cycles were associated with higher euploidy rates than PGT-A cycles (OR: 3.31, *P* < .001), whereas the biopsied blastocyst stage was associated with reduced odds of euploidy (*P* < .1).

**Table 2:** Results of univariable and multivariable analyses using the generalized linear mixed-effects model.

Variable	Univariable analysis		Multivariable analysis	
	OR (95% CI)	*P*-value	aOR (95% CI)	*P*-value
Female age (yr)	0.85 (0.82–0.87)	<.001***	0.88 (0.84–0.93)	<.001***
Duration of infertility (yr)	0.95 (0.90–0.99)	.021**	0.99 (0.95–1.03)	.633
AMH (ng/mL)	1.19 (1.10–1.28)	<.001***	1.1 (1.01–1.2)	.033**
Male age (yr)	0.90 (0.88–0.93)	<.001***	0.98 (0.95–1.02)	.306
Morphologically normal sperm (%)	0.92 (0.86–0.99)	.018***	0.96 (0.9–1.02)	.163
Sperm DNA fragmentation index (%)	0.98 (0.97–0.99)	.008**	1 (0.99–1.01)	.752
E_2_ on hCG trigger day (pg/mL)	1.01 (1.00–1.01)	.082*	0.99 (0.98–1)	.025**
Number of oocytes	1.05 (1.03–1.08)	<.001***	0.99 (0.94–1.04)	.58
Number of MII oocytes	1.07 (1.04–1.10)	<.001***	1.03 (0.96–1.1)	.424
Number of biopsied embryos	1.23 (1.15–1.31)	<.001***	1.05 (0.96–1.14)	.274
Cleavage quality: good vs poor	1.11 (0.81–1.50)	.521	0.84 (0.62–1.15)	.278
PGT-M vs PGT-A	3.31 (2.25–4.85)	<.001***	1.12 (0.73–1.7)	.605
Inner cell mass grade: B vs C	1.09 (0.76–1.57)	.641	1.46 (0.98–2.17)	.065*
Inner cell mass grade: A vs C	2.29 (1.41–3.73)	<.001***	1.69 (1.01–2.82)	.045**
Trophectoderm grade: B vs C	2.36 (1.72–3.24)	<.001***	2.43 (1.71–3.46)	<.001***
Trophectoderm grade: A vs C	5.38 (3.39–8.53)	<.001***	4.42 (2.78–7.04)	<.001***
Biopsies blastocyst stage: D6 vs D5	0.72 (0.53–0.97)	.031**	1.09 (0.8–1.5)	.587
Biopsied blastocyst stage: D7 vs D5	0.50 (0.24–1.06)	.072*	1.28 (0.61–2.71)	.513

AMH = anti-Müllerian hormone, aOR = adjusted odds ratio, CI = confidence interval, E_2_ = estradiol, hCG = human chorionic gonadotropin, MII = metaphase II, OR = odds ratio, PGT = preimplantation genetic testing, PGT-A = preimplantation genetic testing for aneuploidy, PGT-M = preimplantation genetic testing for monogenic disorders.

* *P* < .1.

** *P* < .05.

*** *P* < .001.

**Figure 2.**
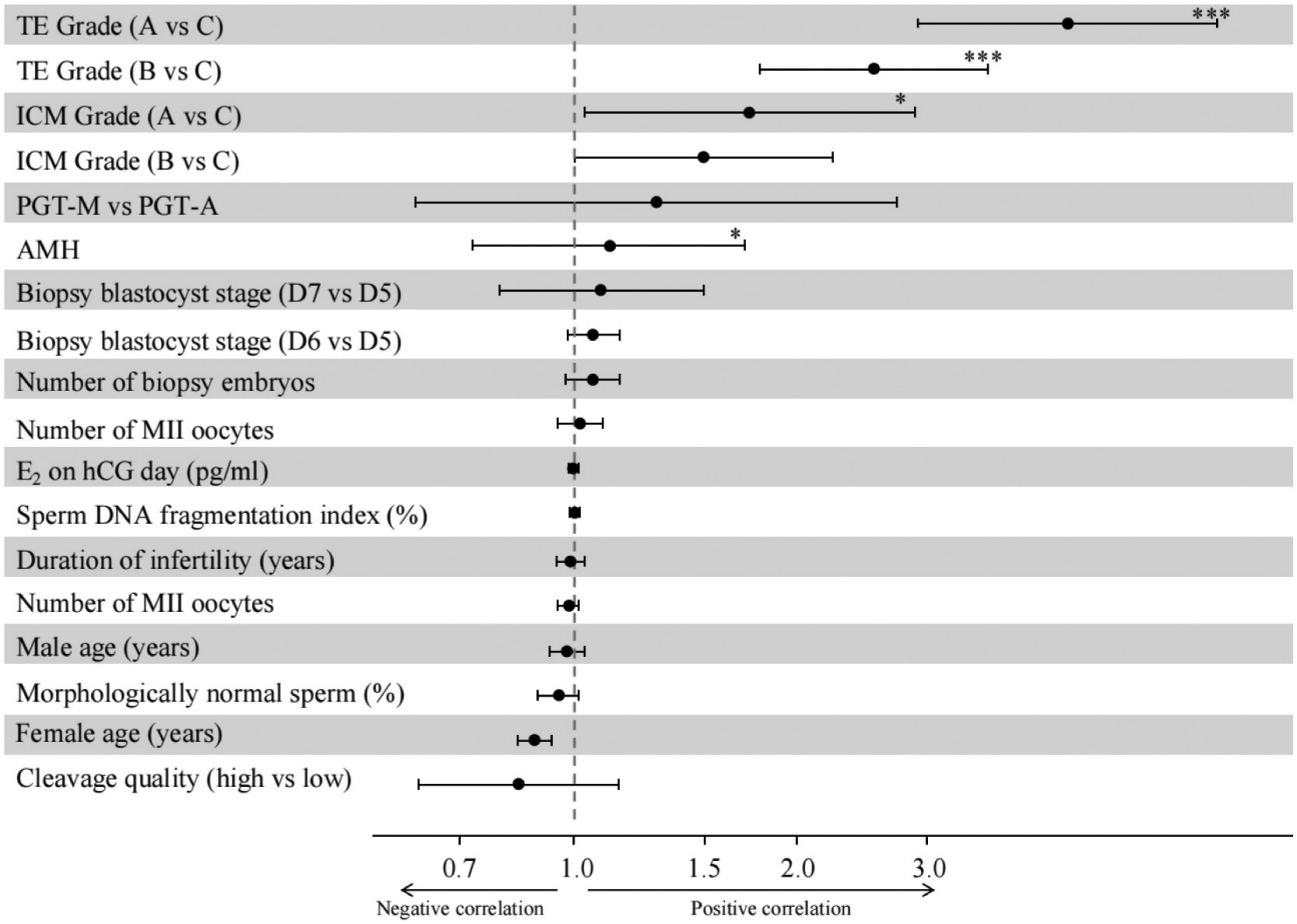
Forest plot of the multivariable analysis of factors associated with embryo euploidy. The horizontal axis represents the odds ratio (log scale). Points to the right of the line indicate variables positively associated with euploid probability, whereas those to the left indicate negative associations. Horizontal bars denote 95% confidence intervals. The vertical axis lists the predictors included in the multivariable generalized linear mixed-effects model. *** *P* < .001; **P < .01; *P < .05. AMH = anti-Müllerian hormone, hCG = human chorionic gonadotropin, ICM = inner cell mass, MII = metaphase II, PGT-A = preimplantation genetic testing for aneuploidy, PGT-M = preimplantation genetic testing for monogenic disorders, TE = trophectoderm.

After adjusting for potential confounders, multivariable GLMM analysis identified 3 significant independent predictors of embryonic euploidy (Table 2, Fig. 2). Female age demonstrated a strong negative association, with each additional year corresponding to a reduced likelihood of euploidy (aOR: 0.88, 95% CI: 0.84–0.93; *P* < .001). AMH levels retained a modest independent positive association with euploidy (aOR: 1.10 per ng/mL; *P* = .033), whereas estradiol concentrations on the hCG trigger day were statistically significant but had a negligible effect size (aOR: 0.99; *P* = .025). Given their small effects, these variables were considered statistically significant but of limited clinical relevance and were not emphasized as key independent predictors. After adjustment, male age, morphologically normal sperm percentage, and DFI were not independently associated with euploidy. Conversely, ICM and TE grades emerged as significant positive morphological predictors. Compared with Grade C, Grade B TE was associated with a greater than twofold increase in the odds of euploidy (aOR: 2.43, 95% CI: 1.71–3.46; *P* < .001), whereas Grade A TE demonstrated an even more robust fourfold increase (aOR: 4.42, 95% CI: 2.78–7.04; *P* < .001). Similarly, although not significant, Grade B ICM was associated with an increase in the likelihood of euploidy (aOR: 1.46, 95% CI: 0.98–2.17; *P* = .065), whereas Grade A ICM demonstrated a significant increase (aOR: 1.69, 95% CI: 1.01–2.82; *P* = .045).

Following multivariable adjustment, no other potential predictors demonstrated a statistically significant independent association. These included cycle performance indicators, such as the number of oocytes retrieved, MII oocytes, cleavage quality, and biopsied embryos, as well as the biopsied blastocyst stage and PGT type.

**Figure 3.**
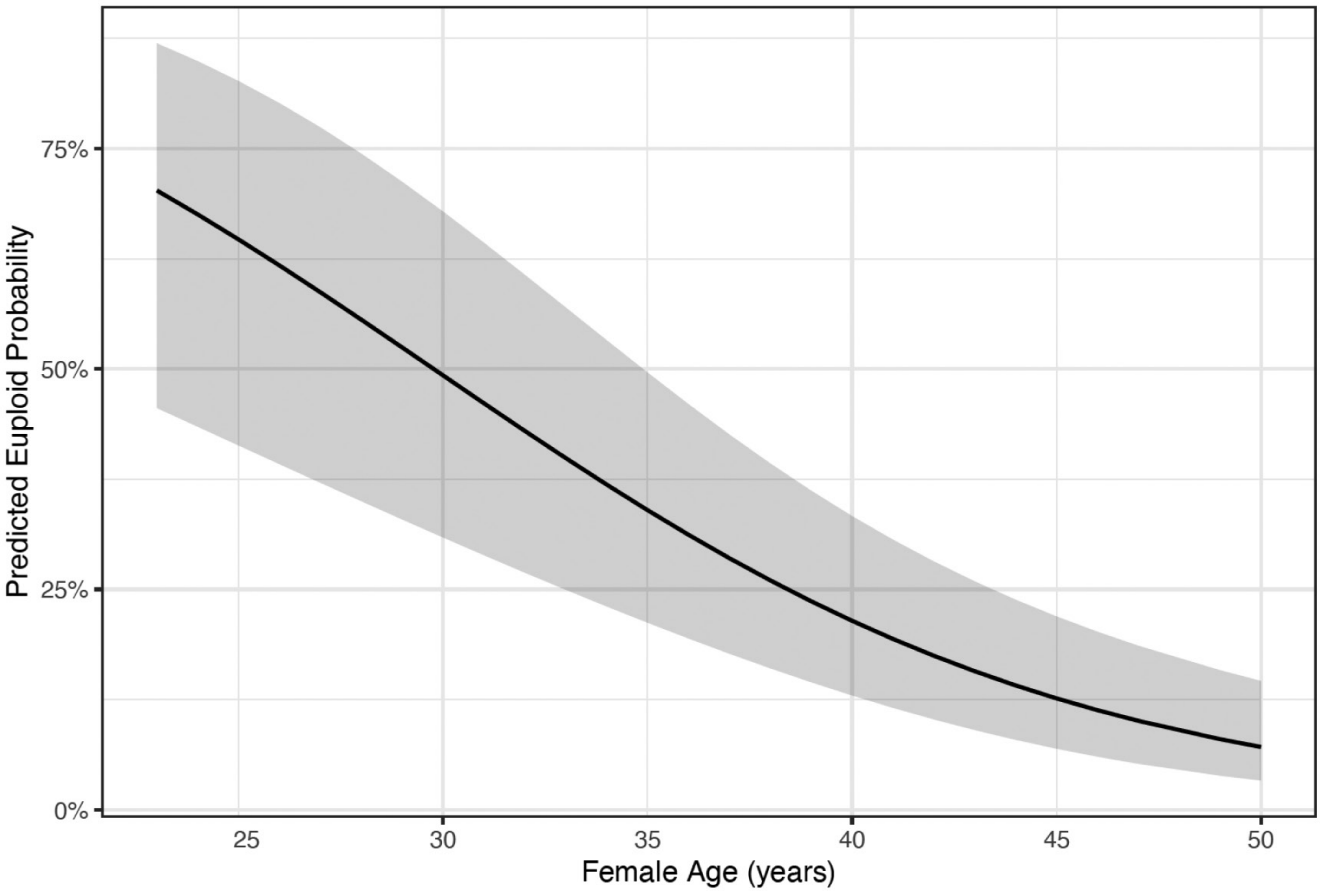
Predicted relationship between female age and embryo euploid probability based on the generalized linear mixed-effects model. The horizontal axis represents female age (years), and the vertical axis represents the predicted probability of euploid embryos. The shaded area indicates the 95% confidence interval of the prediction.

**Figure 4.**
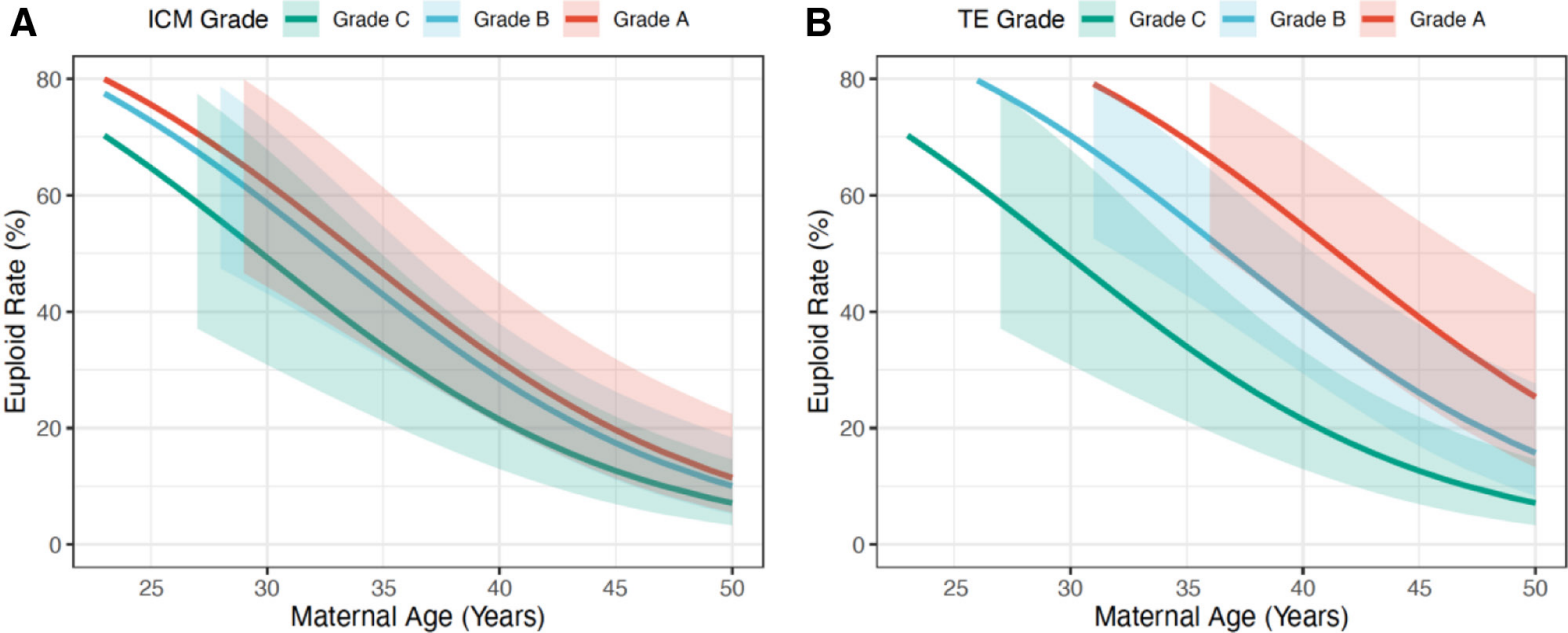
Maternal age–dependent predicted blastocyst euploid rate by inner cell mass (ICM) and trophectoderm (TE) grade. (A) Predicted blastocyst euploid rate by ICM quality and maternal age. (B) Predicted blastocyst euploid rate by TE quality and maternal age. The horizontal axis represents maternal age (years), and the vertical axis represents the predicted euploid rate (%). The curves represent model-predicted probabilities, with shaded areas indicating 95% confidence intervals.

After adjusting for covariates and accounting for random effects, maternal age was significantly negatively associated with the euploidy rate (Fig. 3). At any given age, higher ICM and TE quality grades were associated with higher euploidy rates (A > B > C; Fig. 4).

## 4. Discussion

This retrospective cohort study demonstrated that maternal age and blastocyst morphology, specifically ICM and TE grades, were independent predictors of embryonic euploidy after accounting for within-patient clustering using embryo-level GLMMs.

Across the entire cohort, the overall euploidy rate was 48.1%, consistent with rates reported in previous studies.^[20]^ In our cohort, the euploid group had younger parents, a shorter duration of infertility, and higher AMH levels. They also had higher hCG trigger day estradiol concentrations and greater cycle yields, including more oocytes retrieved, MII oocytes, and biopsied blastocysts. Most conventional semen parameters were similar between the euploid and aneuploid groups; however, the euploid group showed a slightly lower sperm concentration, a lower DFI, and a statistically different distribution of morphologically normal sperm despite identical medians. Embryology metrics favored the euploid group, with higher proportions of high-quality blastocysts and more favorable TE and ICM grade distributions. COH protocol distribution was comparable, whereas PGT type differed, with more PGT-M cycles in the euploid group.

We conducted univariate and multivariate analyses to better elucidate the determinants of embryo euploidy. Given that multiple blastocysts were often derived from the same patient, a blastocyst-level GLMM was applied to account for the nonindependence of observations. Furthermore, our design restricted inclusion to the first PGT cycle per couple, and cycle-level clustering was not observed. By incorporating patient-level random effects, this approach effectively corrected for intra-patient clustering and minimized weighting bias, thereby yielding more reliable and biologically meaningful effect estimates.

In the univariable GLMMs, older maternal and paternal age, higher sperm DFI, a lower percentage of morphologically normal sperm, and lower cycle yields were associated with reduced odds of euploidy, whereas higher AMH levels, higher hCG trigger day estradiol concentrations, better ICM and TE grades, greater cycle yields, and PGT-M (vs PGT-A) were associated with increased odds. In the multivariable GLMM with a patient-level random intercept, maternal age remained a robust independent predictor. The probability of euploid embryos was relatively high up to approximately 30 years of age, declining gradually thereafter, with a sharp decrease after 35 years of age and plateauing at a lower, more stable level beyond 40 years of age (Fig. 2). This finding aligns with the well-established age-related decline in meiotic spindle integrity, cohesin stability, and mitochondrial function, all of which collectively contribute to an increased risk of chromosomal errors with advancing age.^[21,22]^ We observed a strong positive correlation between blastocyst morphology, particularly TE grade, and euploidy rate. Compared with Grade C, Grades B and A TE were associated with significantly higher odds of euploidy (aOR: 2.43 and 4.42, respectively; both *P* < .001), while Grade A ICM was positively associated with euploidy (aOR: 1.69, *P* = .045). These results are consistent with those of a previous study.^[23]^ TE grading likely reflects the proliferative capacity and structural integrity of the outer cell layer of blastocysts, thereby serving as a more sensitive morphological indicator of chromosomal normality. In contrast, the weaker association between ICM grade and euploidy may reflect differences in the biological and morphological information represented by each grading parameter. Further studies are warranted to elucidate these mechanisms.

Furthermore, we analyzed the joint effects of age and morphology on euploid probability (Fig. 3); within each maternal-age stratum, higher ICM/TE grades were associated with higher predicted euploid rates, even though all trajectories showed an age-related decline that accelerated beyond the mid-30s and stabilized at a lower level after 40 years of age. The dynamic range is larger for TE than for ICM, aligning with the multivariable GLMM in which TE grades had stronger stepwise associations with euploidy than ICM, supporting TE as the more sensitive morphological indicator of chromosomal normality. Clinically, these findings suggest that morphology can refine embryo selection at any age, although it cannot fully offset the age-driven decline in euploidy and should therefore be integrated with age-specific counseling and prioritization strategies.

AMH levels exhibited a modest but independent positive association with euploidy (aOR: 1.10, *P* = .033). As a marker of ovarian reserve, AMH may enhance the probability of obtaining euploid embryos, primarily by increasing the number of available oocytes rather than by directly influencing the chromosomal competence of individual embryos. Although estradiol levels on the hCG trigger day remained statistically significant in the multivariate model, the effect size was negligible (aOR: 0.99, *P* = .025), suggesting limited clinical relevance. Estradiol levels likely affect euploidy indirectly by increasing the number of viable oocytes and embryos available for selection, rather than directly determining chromosomal integrity. Similarly, the number of retrieved oocytes, MII oocytes, and biopsied blastocysts was significant in univariable analyses but lost significance after controlling for maternal age, AMH, and morphology, indicating that these variables are not independent determinants. Their primary clinical value lies in expanding the embryo selection pool.

The distribution of COH protocols did not differ between euploid and aneuploid groups, and COH was not retained as an independent predictor, indicating that stimulation strategy does not alter embryonic chromosomal competence. Taken together, these findings do not support an iatrogenic increase in aneuploidy attributable to IVF stimulation or cycle management and instead align with the theory that stimulation parameters improve the chance of obtaining at least 1 euploid blastocyst by increasing yield, consistent with previous evidence showing no material effect of stimulation protocol or intensity on blastocyst euploidy.^[24]^

Moreover, cleavage-stage (D3) embryo morphology grading was not significantly associated with subsequent blastocyst euploidy. This suggests that the morphology of D3 embryos alone is insufficient to predict chromosomal status. Notably, some D3 embryos with suboptimal morphology were still able to develop into euploid blastocysts, consistent with previously reported findings.^[25]^ During the first D3 stage, the survival of embryos with severe forms of aneuploidy may depend largely on oocyte-derived proteins; however, beyond the 4- to 8-cell stage, embryos must increasingly rely on their own genome activation.^[26]^ Therefore, aneuploidy may exert a more pronounced impact beyond D3, particularly during the blastocyst stage. This finding supports the practice of extended embryo culture in ART, as it helps avoid premature exclusion of embryos that may retain developmental potential. Therefore, extending culture to the blastocyst stage may increase the likelihood of obtaining a transferable euploid embryo, particularly in patients with a diminished ovarian reserve or limited oocyte yield.

Male-related variables were not independent determinants of euploidy after adjustment in our cohort, despite inverse univariable associations for morphology and DNA fragmentation, suggesting confounding by maternal age, ovarian response, and blastocyst morphology. Nevertheless, prior studies have reported that paternal age and semen parameters can influence blastulation and euploidy, indicating a potential male contribution to embryo quality beyond what is captured by routine metrics.^[12]^ This apparent discrepancy underscores the limited sensitivity of conventional semen indices and global DFI measures for predicting chromosomal competence in the embryo; supported by reports showing no material effect of DFI on euploidy in specific subgroups.^[27]^ Future work should evaluate more granular and functional male biomarkers and selection approaches.

The findings of our study should be interpreted in consideration of certain limitations. First, due to the observational nature of the study design, we can identify associations but cannot establish causality between the predictors and euploidy. Second, although we utilized multivariable GLMMs to adjust for known covariates, the potential for residual confounding remains. Third, selection bias cannot be entirely ruled out, given the single-center design and the exclusive inclusion of PGT cycles, which represent a specific patient population with potentially distinct prognostic profiles compared to the general IVF population. Finally, although embryonic euploidy is an informative intermediate endpoint essential for transfer selection, live birth remains the ultimate goal of ART. Future multicenter studies with larger sample sizes are warranted to link predictive factors for euploidy with cumulative live birth outcomes, thereby strengthening their clinical relevance and applicability.

## 5. Conclusion

This study identifies maternal age and blastocyst morphology as the most significant independent predictors of embryo euploidy in PGT cycles. Notably, ovarian stimulation parameters and oocyte yield showed limited association with euploidy rates after adjusting for confounders. These findings suggest that embryonic chromosomal competence is likely a reflection of intrinsic oocyte quality and developmental potential rather than a direct consequence of the stimulation protocol employed. Therefore, integrating morphology-based assessments with age-specific considerations may improve the precision of embryo selection and patient counseling in ART.

## Acknowledgments

We are grateful to all participants involved in this study and would like to thank Editage (www.editage.cn) for their professional assistance in English language editing.

## Author contributions

**Conceptualization:** Hui Huang, Fengxia Liu, Xueyuan Liu.

**Formal analysis:** Kali Huang, Li Deng, Xuefei Liang.

**Writing – original draft:** Fengxia Liu.




