# Supplementary material for: Maternal age and blastocyst morphology as independent predictors of embryonic euploidy in preimplantation genetic testing cycles: A retrospective cohort study
Source: Medicine (Baltimore). 2026 Jun 5;105(23):e49126. doi: 10.1097/MD.0000000000049126 (PMC13246117; doi:10.1097/MD.0000000000049126)
Supplement: Supplementary file 1 [file medi-105-e49126-s001.docx]

Supplementary Table 1

Table S1. Variable definitions and data types used in statistical analyses

| **Feature** | **Type** | **Categorisation** |
| --- | --- | --- |
| **Female age (years)** | Numeric |  |
| **Female BMI (kg/m^2^)** | Numeric |  |
| **Duration of infertility (years)** | Numeric |  |
| **AMH (ng/mL)** | Numeric |  |
| **Basal FSH (IU/L)** | Numeric |  |
| **Male age (years)** | Numeric |  |
| **Male BMI (kg/m^2^)** | Numeric |  |
| **Sperm concentration (×10^6^/mL)** | Numeric |  |
| **Sperm viability (%)** | Numeric |  |
| **Progressive motility** | Numeric |  |
| **Non-progressive motility** | Numeric |  |
| **Morphologically normal sperm (%)** | Numeric |  |
| **Sperm DNA fragmentation index (%)** | Numeric |  |
| **E_2_ on hCG day (pg/mL)** | Numeric |  |
| **Number of oocytes** | Numeric |  |
| **Number of MII oocytes** | Numeric |  |
| **Number of biopsied embryos** | Numeric |  |
| **COH protocol** | Categorical | Long/antagonist protocol |
| **PGT type** | Categorical | PGT-A/PGT-M |
| **Ploidy status** | Categorical | Euploidy/aneuploidy |
| **Cleavage-stage embryo quality** | Categorical | Low/high quality |
| **Biopsied blastocyst quality** | Categorical | Low/high quality |
| **Inner cell mass grade** | Categorical | A/B/C |
| **Trophectoderm grade** | Categorical | A/B/C |
| **Biopsied blastocyst stage** | Categorical | D5/D6/D7 |

Abbreviations: AMH, anti-Müllerian hormone; BMI, body mass index; COH, controlled ovarian hyperstimulation; E_2_, oestradiol; FSH, follicle-stimulating hormone; hCG, human chorionic gonadotrophin; MII, metaphase II; PGT, preimplantation genetic testing; PGT-A, preimplantation genetic testing for aneuploidy; PGT-M, preimplantation genetic testing for monogenic disorders.
