# Supplementary material for: Maternal age and blastocyst morphology as independent predictors of embryonic euploidy in preimplantation genetic testing cycles: A retrospective cohort study
Source: Medicine (Baltimore). 2026 Jun 5;105(23):e49126. doi: 10.1097/MD.0000000000049126 (PMC13246117; doi:10.1097/MD.0000000000049126)
Supplement: Supplementary file 2 [file medi-105-e49126-s002.pdf]

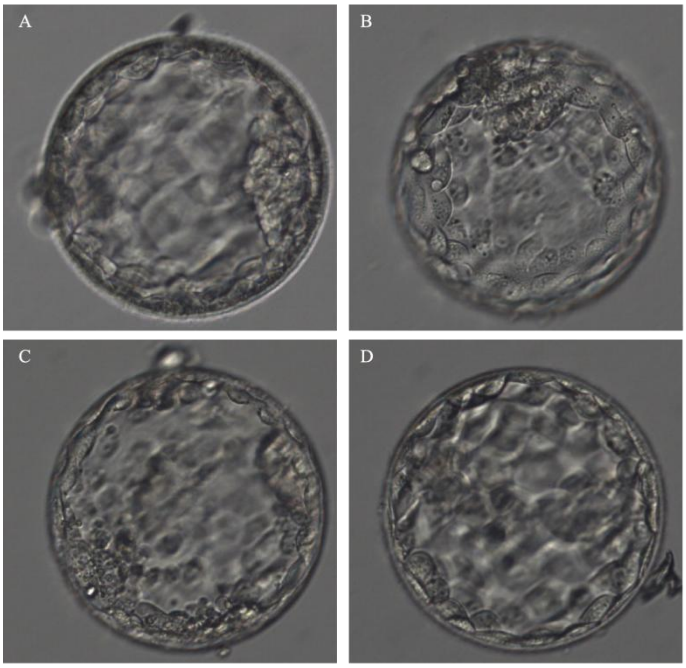

Supplemental Digital Content 2. Representative images of blastocysts with different morphological qualities. The figure displays blastocysts with varying grades according to the Gardner scoring system: (A) Grade 4AA; (B) Grade 4BA; (C) Grade 4BC; and (D) Grade 4CA.
